# Migraine*Read before the Buffalo Medical Club, Aug. 18, 1880.

**Published:** 1880-12

**Authors:** Thos. Lothrop


BUFFALO

MEDICAL AND SURGICAL JOURNAL.

VOL. XX.—DECEMBER, 1880.—No. 5.

### Original Communications.

### MIGRAINE.*

BY THOS. LOTHROP, M. D.

THE word Migraine, used by the French, and Megrim of our own vernacular, have a common Greek derivation, and are employed by the neurologist to designate a variety of neuralgia, usually considered to be trigeminal, one of the most common forms of this very prevalent disease. It is selected as an important subject for investigation and inquiry, especially so in view of its alarming frequency during the period of bodily development, when the system is under the strain of excessive mental work. We may regard the present faulty system of female education, in which the nervous system, especially prior to puberty, is exercised to an extent disproportionate to its powers of endurance, while the muscular, the digestive, and the vascular functions are so far neglected that they fail to impart the vigor required to maintain the body in a healthy equilibrium, as an important causative element in this and other varieties of the neuroses. The increasing frequency of nervous disorders in our

* Read before the Buffalo Medical Club, Aug. 18, 1880.

American life, embraces a field for professional study, which has brought forth some of the best contributions to recent medical literature.

In discussing migraine, the writer is compelled from necessity to treat it in a practical way, avoiding as far as possible useless and vague theories, while attempting to show what is known of its etiology, and also what can be done for its relief.

Pre-eminently migraine is an inherited disease. This may be more positively stated than in regard to any other of the neuroses. A case has fallen in my own experience of a mother who transmitted to her offspring this legacy of suffering she herself had unfortunately inherited. A young medical friend, the victim of this disease, traces its origin to his mother, who is the subject of severe trigeminal neuralgia, which she inherited from an epileptic parent. The reciprocal relation existing between migraine and epilepsy has been taken as an argument to prove the identity or similarity of causes, and of lesions giving rise to the two diseases. Assuming that migraine is a form of trigeminal neuralgia, Anstie, Sequin, and others conclude that a lesion exists in those portions of the Pons varolii and medulla oblongata, which give rise to the sensory roots of the trigeminus. This centric origin of the disease, it seems to me, is more easily assumed than clearly demonstrated, and yet its analogy to epilepsy in many of its features points to the great nerve centres as its source and origin. The best authority on the etiology and pathology of neuralgia, leads the inquirer into a network of finely spun theories, from which we escape but little the wiser for the venture. This want of positive knowledge in regard to the causes of many of the neuroses leads to indefiniteness in nomenclature, and also to great difficulty in differential diagnosis. In proof of this we may cite the fact that many writers use as synonymous terms hemicrania and migraine, and describe the symptoms and phenomena observed in this variety of neuralgia under one or both of these terms, while the disease under consideration is often, indeed most frequently, termed trigeminal neuralgia.

In endeavoring to ascertain the causes of this form of neuralgia, it seems to me that the function of the spinal cord, simply in the transmission of reflex-sensations, is too little considered, and that too great stress is placed upon its centric origin. It is acknowledged that a peculiar susceptibility to reflex action prevails in certain temperaments, while in others, all possible reflex sensations are difficult and often impossible to produce. The cause of this is as difficult of explanation as any other individual idiosyncrasy. The trigeminus, either on account of its origin, or of its distribution, clinically is known as a frequent avenue for the transmission of reflex sensations, conveyed to and through the spinal cord from a variety of sources in the organism. The reason for this is also not clear. We may conjecture that the ganglionic cells of that portion of the medulla from which the trigeminus takes its origin, possess a peculiar susceptibility to the reception of impressions, and therefore the most common avenue for the transmission of such impressions. In the pathology of this and other neurotic diseases, we cannot fail to recognize the important position of reflex action. In migraine, whether its origin is traced to a spinal lesion, as the primary seat of the disease, or whether such lesion, if any exist, is secondary, due to the effect of constant excitation upon the ganglionic cells of the medulla, such excitation primarily arising from morbid processes at the periphery of nerves, or whether it is really a neurosis of the sympathetic nerve, there is a broad field for scientific inquiry, which is at present imperfectly understood.

Sequin points to the similarity of symptoms and conditions in migraine and epilepsy, and conjectures the centric origin of migraine. It is known that cases of migraine have finally resulted in epileptiform seizures. Brown-Sequard, as early as 1850, called attention to the relation of lesions of the spinal cord and certain spinal nerves to epilepsy, while Van der Kolk in an exhaustive work, which is included in one of the early volumes of the new Sydenham Society publications, demonstrated in epilepsy a lesion of the posterior half of the medulla. Contraction of the vessels of the cerebral pia mater has also been observed by Brown-Sequard in epileptic animals, while others have observed that irritation of the peripheral nerves produces a like contraction of the cerebral vessels which explains epileptic seizures under the influence of peripheral irritation. This may be termed reflex epilepsy, and in the lighter forms, epileptic vertigo or petit mal. The occurrence of the aura of migraine, similar to the aura of epilepsy, is an interesting coincidence in considering these two varieties of the neuroses, while the periodical return of attacks of migraine, similar to that observed in epilepsy, is also deserving of note.

These subjective conditions, giving rise to these two diseases are referred to in this connection to explain a position, which, while it has not been established by actual experiment and investigation, may be inferred from the premises we have laid down: that is, inasmuch as it is established that epilepsy is due to a spinal lesion, and there exists a similarity of symptoms in the two diseases, we may be justified in saying that in *well-established* cases of migraine there will be found a like condition of the ganglionic cells of the medulla. I think this inference is justified by the facts, which have been imperfectly presented. Further investigations in pathology of the neuroses will, I do not hesitate to say, fully corroborate this view.

Migraine also is essentially one of the hystero-neuroses. The sexual organization of woman is the source of a series of nervous phenomena, which, while they are heterogeneous in their manifestations, are closely allied in their etiology. A pathological condition of the uterus and its appendages in which we include the ovaries, is a fruitful source of reflex impressions. The trigeminus and sympathetic nerves are frequent avenues for the transmission of these impressions to and from the spinal cord and medulla. But little is found in medical literature upon this subject. Fordyce Barker, in his work "On the course and treatment of reflex insanity in woman," demonstrates the influence of the uterus in its periodical menstrual changes, in its pregnant condition, as well as during the period subsequent to parturition as a causative element in the production of mental aberration. In the causation of the neuralgias there exists a close and undisputed relation, borne out by facts, occurring in daily professional experience. The most severe cases of migraine met with in practice are connected with the menstrual epoch.

The relative proportion of cases in the sexes is, according to Eulenberg, as five in the female to one in the male. This predisposition of females to the disease is a fact deserving of consideration, and points to the menstrual function as among the most frequent of the probable causes. The occurrence of attacks of migraine in females, who are not subjects of dysmenorrhea, and in whom the uterus, from the most careful examination, fails to unfold any organic lesion, is a problem for the neurologist to solve. Cases are not infrequent in which at every menstrual period, migraine, is as certain as the menstrual flow itself, and the victim suffers, not from the local congestion or hyperamia, incident to this peculiarity of her sex, but from reflex sensory impressions which have been transmitted from a distant part of the organism, to the head, only to increase the torture, which would be more tolerable if confined to its seat of origin.

It would weary your patience, and prove too great a tax upon my time, to attempt to notice the various causes of migraine, and their relations to local and constitutional conditions. Enough has been said to direct attention to the more prominent and important conditions. Neither would it be profitable to refer to its symptomatology, which must be familiar to all. The importance of therapeutical indications to the practitioner so far transcend all other questions, when called to the bedside of a victim suffering from this disease, that the writer begs to direct attention to its treatment, with hasty reference to such cases as may be of practical utility to members of the Club.

The principles enunciated above afford a natural subdivision for the treatment

1st. Treatment of the prodromata.

2d. Treatment of the attacks.

3d. Treatment of the pathological condition of the nervous system upon which migraine depends.

1st. Plainly if the migraine depends upon atonic dyspepsia, the use of nux vomica in small doses, as for instance, one drop of the tincture every hour during the waking hours, combined with tincture of belladonna in half or fourth-drop doses, will prove of benefit. Sequin also recommends the patient to reduce the saccharine and amylaceous foods. In cases depending upon anæmia, depraved nutrition, tonics of iron and cod liver oil are indicated.

2d. Treatment of the attack. If depending upon the presence of undigested food in the stomach, a good emetic is the speediest and most effectual way to relieve the sufferings; avoid all food during the attack; give the stomach rest; an exception to the rule may be made in cases in which the patient strongly desires some particular article. I have a case on hand in which dried beef has "touched the spot," to use the words of the sufferer; another in which a few strawberries would satisfy the stomach. These are only suggestive, and afford a practical hint which may be useful. I have also known a few teaspoonfuls of whiskey to afford relief. There can be no doubt of the efficacy of guarrana in the early period of the attack. The fl. ext. in doses of two teaspoonfuls every half hour, until three or four doses have been given, especially if given before nausea sets in, has often proved beneficial in my hands, and as often failed. It is deserving of a trial.

Another and more recent remedy is found in the nitrite of amyl. Eulenberg (Ziemmsen's Cyclo., vol. xiv, p. 27) states that "the indications for its use depend on the fact that it possesses the power of dilating the blood vessels, although whether by acting on the contracted elements or by paralyzing the vaso-motor system is yet unsettled." Dr. Berger, in the *Medical Times and Gazette*, (1870, II, p. 469) first employed this agent in a case of migraine with almost instant effect; "the pain was charmed away" as it were, and did not return during the day. Holst states that in a female patient the attack itself was not only cut short, but its recurrence was postponed longer than usual.

Dr. Geo. M. Beard recommends caffein in 2 grain doses, to be repeated every hour until 3 or 4 doses have been given, and claims for it positive merits.

Chloral hydrate is also recommended in doses of 10 to 15 grains, but fails in my hands.

But the surest remedy is morphia administered hypodermically. In doses of $\frac{1}{4}$ grain combined with $\frac{1}{50}$ to $\frac{1}{60}$ grain of atropia, it is par excellence the remedy in the severer forms of migraine. I especially direct attention to the combination of morphia and atropia. The use of the latter remedy is very important. The reason is found in the similarity of migraine to epilepsy, in its pathological relations. A large experience in the use of these remedies in the severer forms of migraine, has convinced me of their necessity and great utility. It is useless to waste time in any other direction. I am now referring to the more violent forms. In the mild cases, I would not advise its use, other and safer remedies can be substituted, and in the majority of cases the disease made to yield.

3d. The treatment of migraine as a pathological entity, with a view to its cure, is a most important matter. Reference has been made above to the lesion of the medulla, upon which migraine is supposed to depend, and upon its similarity in some of its features to epilepsy, with a view to direct attention to the therapeutical indications. Lesions of the spinal cord, and especially of its cranial portion, have generally been found intractable to treatment, and efforts towards their alleviation and cure have not been attended with results at all satisfactory to the profession. The introduction of the bromides has worked a marvelous change in the treatment of epileptiform disease since Van der Kolk gave to the profession a rational idea of its pathology. The system he recommended of prolonged blistering near the occiput, from which he claimed excellent results, has been supplanted entirely by the bromides, to which belladonna and its alkaloid, atropia, have become valuable auxiliaries. What the bromides and belladonna are to Epilepsy, cannabis indica is to migraine; not that either of these medicinal agents or any combination of them will cure every case that may come under observation, but that they will relieve many, and even such as are not dependent upon severe spinal lesions, is now generally accepted by the profession.

In 1872, in a short article, contributed to the *London Practitioner*, Dr. Richard Greene brought out Cannabis Indica as the important remedy for migraine. Prof. Sequin, in the *Medical Record*, (vol. XII, page 774, 1877,) in an excellent article, confirms the experience of Dr. Greene in the use of this agent, and substantiates his view by the experience of other eminent medical men.

The principle of treatment laid down by Dr. Greene, is to maintain by the use of small doses of the agent, a constant influence upon the nervous system for a long time, the same as is required in epilepsy by the use of the bromides. At first, as a matter of course, no appreciable effect is observed, and not until the use of the remedy is persevered in for many weeks, and the nervous system kept under its influence for a considerable time, will the patient find an appreciable diminution in the severity and frequency of the attacks. It is well to commence with one-fourth grain of the extract, before each meal, for the first fortnight. The dose may be increased to the third of a grain for the second fortnight, to be augmented to a half grain, at the end of four weeks. This amount will generally be sufficient and should be faithfully continued for several months. Success here is only obtained by persevering effort. Failure is often complained of, when on inquiry, the agent has not had a fair trial; and to this want of perseverance in other diseases, besides the one under consideration, may justly be ascribed many of discouragements which attend the well-directed efforts of painstaking and conscientious practitioners.

The writer would not have trespassed upon the time and patience of the Club, if the facts and principles above enunciated were not borne out in his own practice. A case is in hand in which hereditary influences bore a prominent part in its causation; in which the skill of the most eminent men in the metropolis had failed to afford any relief, the patient finally resigning herself to the suffering which seemed inevitably to be entailed upon her at each menstrual epoch, the only hope of relief being in the approach of the climacteric which was many years in the future. Hemicrania in its severest form, with nausea, insomnia always followed each menstruation. Life was indeed burdened with the anticipation fulfilled with never-varying certainty of two or three days in each month of suffering from which there seemed no escape, and hence no relief. The prolonged use of cannabis indica for the period of one year, has afforded such relief that the nervous system has had time to regain long-lost vigor, and the patient is in better health than for many years. Other cases might be cited confirmatory of the utility of the agent. Is the question asked, Has the remedy ever failed in my hands? and I can answer that it has not in any case in which its prolonged use has been made. The trouble is in the want of perseverance of the patient, not in the efficacy of the remedy. I venture to say, however, that in cases due to an aggravated lesion of the medulla, in subjects of feeble nutrition, and of an exhausted and worn-out nervous system, there could not be expected relief from any remedy. Physicians cannot re-mould their patients, nor infuse into their exhausted organizations a larger measure of vital force than they were endowed with at their birth. Migraine, or any other disease, in such subjects are beyond the reach of medical skill; but in well-selected cases, indeed, in the majority of cases, if we do not succeed in affording permanent relief for all, we certainly will diminish the severity of the attacks, and the frequency of their recurrence, just as surely as we accomplish this end in epileptiform seizures by the use of bromide and belladonna.

This contribution, for which an apology is due the Club for its imperfections on account of the haste in which it has been prepared, and also on account of the paucity of information in medical literature, especially in regard to migraine, is hesitatingly presented with the earnest wish that it may lead its members to investigate a most interesting type of disease, and also to the use of the important remedy here suggested.

